# Indigenous Peoples and research: self-determination in research governance

**DOI:** 10.3389/frma.2023.1272318

**Published:** 2023-11-15

**Authors:** Ibrahim Garba, Rogena Sterling, Rebecca Plevel, William Carson, Felina M. Cordova-Marks, Jewel Cummins, Caleigh Curley, Dominique David-Chavez, Adam Fernandez, Danielle Hiraldo, Vanessa Hiratsuka, Maui Hudson, Mary Beth Jäger, Lydia L. Jennings, Andrew Martinez, Joseph Yracheta, Nanibaa' A. Garrison, Stephanie Russo Carroll

**Affiliations:** ^1^Lands of the O'odham and Yaqui peoples, Mel and Enid Zuckerman College of Public Health, University of Arizona, Tucson, AZ, United States; ^2^Lands of the O'odham and Yaqui peoples, Native Nations Institute, Udall Center for Studies in Public Policy, University of Arizona, Tucson, AZ, United States; ^3^Living on the lands of Waikato-Tainui, Te Kotahi Research Institute, University of Waikato, Hamilton, New Zealand; ^4^Lands of the Congaree, Catawba, Muscogee, and Eastern Cherokee, Law Library, School of Law, University of South Carolina, Columbia, SC, United States; ^5^Lands of the O'odham and Yaqui peoples, American Indian Studies-Graduate Interdisciplinary Program, College of Social and Behavioral Sciences, University of Arizona, Tucson, AZ, United States; ^6^Nunt'zi (Ute), Hinono'eino' (Arapaho), and Tsitsistas (Cheyenne) homelands, Department of Forest and Rangeland Stewardship, Colorado State University, Fort Collins, CO, United States; ^7^Ancestral homeland of Eastern Siouan-speaking Indigenous peoples (Yesàh, “The People”), American Indian Center, University of North Carolina at Chapel Hill, Chapel Hill, NC, United States; ^8^Dena'ina Ełnena, Center for Human Development, College of Health, University of Alaska Anchorage, Anchorage, AK, United States; ^9^Lands of the Oceti Sakowin (Seven council fires of the Lakota/Nakoda/Dakota), Native BioData Consortium, Eagle Butte, SD, United States; ^10^Ancestral homelands of the Paskestikweya (Piscataway) band of Chaptico, the Moyaone, Nanjemoy, and the Potapoco, Bloomberg School of Public Health, Johns Hopkins University, Baltimore, MD, United States; ^11^Traditional, ancestral and unceded territory of the Gabrielino/Tongva peoples, Institute for Society and Genetics, College of Letters and Sciences, University of California, Los Angeles, Los Angeles, CA, United States; ^12^Traditional, ancestral and unceded territory of the Gabrielino/Tongva peoples, Institute for Precision Health, David Geffen School of Medicine, University of California, Los Angeles, Los Angeles, CA, United States; ^13^Traditional, ancestral and unceded territory of the Gabrielino/Tongva peoples, Division of General Internal Medicine and Health Services Research, David Geffen School of Medicine, University of California, Los Angeles, Los Angeles, CA, United States

**Keywords:** Indigenous, Data Sovereignty, data governance, CARE Principles, self-determination, research governance, research practice

## Abstract

Indigenous Peoples are reimagining their relationship with research and researchers through greater self-determination and involvement in research governance. The emerging discourse around Indigenous Data Sovereignty has provoked discussions about decolonizing data practices and highlighted the importance of Indigenous Data Governance to support Indigenous decision-making and control of data. Given that much data are generated from research, Indigenous research governance and Indigenous Data Governance overlap. In this paper, we broaden the concept of Indigenous Data Sovereignty by using the CARE Principles for Indigenous Data Governance to discuss how research legislation and policy adopted by Indigenous Peoples in the US set expectations around recognizing sovereign relationships, acknowledging rights and interests in data, and enabling Indigenous Peoples' participation in research governance.

## 1. Introduction

The right of Indigenous Peoples to govern themselves as political communities preexisted the creation of the colonial societies and modern nation-states that continue to dictate their collective affairs. Starting in the early twentieth century, acknowledgment of the right to collective self-governance for “peoples” generally has grown globally through norms, policies, and laws centered around the principle of self-determination (League of Nations, 1919; United Nations, 1945; United Nations General Assembly, 1966a,b, 2007; Smith, 2012). We use ‘Indigenous Peoples,' a recognized legal term that affirms their political and rights-based statuses, rather than ‘Indigenous communities' or ‘tribes' (we make an exception for ‘tribe' in citing deidentified documents). Indigenous Peoples' right to self-determination—their right to freely choose and pursue economic, social, and cultural development goals—was recognized internationally in the 2007 United Nations Declaration on the Rights of Indigenous Peoples (UNDRIP) (United Nations General Assembly, 2007). However, this right to self-determination is limited for Indigenous Peoples who share geographies with nation-states in the absence of a corresponding state obligation to recognize and implement Indigenous self-determination in mainstream governance norms and institutions across multiple sectors (United Nations General Assembly, 2007), research included. Accordingly, we view research sovereignty as a form of Indigenous self-determination concerned with securing Indigenous Peoples' control over acquisition, use, storage, and reuse of their data not only under their authority but especially in the custody of researchers and research institutions, e.g., funders, sponsors, repositories, publishers (AIATSIS, 2020; Carroll et al., 2021).

In this paper, we elaborate on Indigenous Data Sovereignty (IDSov) concepts through a discussion of the CARE Principles for Indigenous Data Governance: **C**ollective benefit, **A**uthority to control, **R**esponsibility, and **E**thics (Carroll et al., 2020). Specifically, we focus on “Authority to control,” which addresses the aspirations of Indigenous Peoples to extend their inherent right to self-determination over research data, especially in an era of increasing digitization and novel forms of sharing information. We examine legislation, policy, and processes adopted by Indigenous Peoples in the United States (US) that set expectations around recognizing sovereign relationships, acknowledging Indigenous rights and interests in data, and fortifying Indigenous authority in research governance. We then offer recommendations for Indigenous Peoples, individual researchers, and research institutions to align governance and stewardship practices with Indigenous research and data sovereignty.

## 2. Indigenous research governance and “Authority to control”

IDSov refers to the right of Indigenous Peoples to govern how data from or about them is collected, accessed, used, stored, and disposed of—thereby repositioning Indigenous authority over Indigenous data away from an extractive, colonial model (Kukutai and Taylor, 2016; Carroll et al., 2019; Walter et al., 2021). Indigenous Data Governance (IDGov) entails the nuts and bolts of implementing IDSov: practical issues concerning who decides how and when Indigenous data are accessed, used, and shared (Walter et al., 2018). IDGov and Indigenous research governance overlap; some data are research-derived and are subject to both data and research governance.

Today, Indigenous Peoples continue to develop their research governance infrastructures by harnessing their own ways of knowing, doing, and being. Indigenous Peoples set agendas, determine methodologies, control processes, and direct outcomes of their research ecosystems (Smith, 2012; Hudson et al., 2020). They extend oversight outside their communities by ensuring that their expectations are applied by individuals and institutions through protocols and practices that uphold Indigenous research governance. In other words, Indigenous research governance is an exercise of CARE's “Authority to control” Principle in both Indigenous and non-Indigenous research environments.

## 3. Assertions of Indigenous research sovereignty in the US

Indigenous sovereignty is inherent and not granted through legal recognition by external entities, whether national or international. However, due to varying colonial practices and legal regimes, Indigenous Peoples globally have had to re-exert their sovereign rights from positions of *de jure* (from the law) sovereignty (i.e., explicit status) or *de facto* (in practice) sovereignty (i.e., implicit status). While explicit or implicit status does not determine how Indigenous Peoples exercise sovereignty within their communities, it does impact how they engage external entities (e.g., governments, non-profits, corporations, research institutions) that collect and use Indigenous data. Hence, Indigenous Peoples' explicit or implicit status shapes strategies for exercising their “Authority to control” their data, particularly when used by external actors.

There are currently 574 distinct Indigenous Peoples in the US with explicit status under the federal recognition framework, while over 60 maintain such status with states (National Conference of State Legislatures, 2016; US Bureau of Indian Affairs, 2023). Other Indigenous Peoples have implicit status, with a subset of those seeking explicit status with either federal or state authorities. Indigenous Peoples, especially those without explicit status, face major barriers to exercising their inherent rights and engaging in areas of interest due to limited administrative capacity and access to resources as well as lack of formal recognition by federal and state governments.

Indigenous Peoples in the US have established various review mechanisms to approve research in their communities and with their citizens (Around Him et al., 2019; Kuhn et al., 2020). These governance mechanisms can review research generally or in particular fields (e.g., health, environmental issues, culture). They can be specialized bodies operating with relative autonomy from elected Indigenous leadership or embedded in Indigenous government agencies. In rare cases, they can be an administrative process without a specialized committee to assess proposals. Although not discussed here, they can also be placed in Indigenous academic institutions or regional/inter-Indigenous organizations that assess proposals on behalf of member governments.

Drawing on previous content analysis of research legislation and policy from 26 Indigenous Peoples in the US (Carroll et al., 2021), we illustrate how these official documents detail Indigenous assertions of self-determination in research (Supplementary Table 1). These publicly available documents were obtained from both official Indigenous and third-party online sources, though a number has since become unavailable due to dead or retired links. We anonymize document references within the manuscript based on the governance distinction between accessing publicly available documents and using the documents in analysis or for publication (see Supplementary Table 2 for a complete list of the documents). This practice is informed (1) by conversations with Indigenous research governance professionals and (2) by an analogous distinction we observed in the documents between approval of research projects as a whole and separate approvals for publishing data and/or findings.

We included documents if their review processes were direct exercises of research sovereignty. For instance, we do not discuss Indian Health Service (IHS) IRBs because IHS is a federal, not Indigenous, agency. Also excluded are IRBs at Indigenous research institutions if the body does not review projects on behalf of the entire community.

Guided by the CARE Principle of “Authority to control” and two of its sub-principles—(1) “Recognizing rights and interests” and (2) “Governance of data”—we discuss four thematic areas that demonstrate Indigenous research sovereignty. Under “Recognizing rights and interests” are (i) Sovereignty and self-determination, and (ii) Assessment of collective risks and benefits. Under “Governance of data” are (i) Jurisdiction and control, and (ii) Enforcement.

## 4. Governance for Indigenous research sovereignty

As noted earlier, Indigenous Peoples employ a variety of governance measures (laws, policies, processes, practices) to oversee self-determined research. Recognizing Indigenous Peoples' “Authority to control” research requires (1) respecting Indigenous Peoples' rights and interests in research and data, and (2) creating data governance mechanisms in external institutions that reflect Indigenous Peoples' expectations. Here, we discuss Indigenous expectations expressed in research governance measures as well as resultant obligations for individual researchers and institutions as they develop mechanisms and practices that uphold Indigenous sovereignty while supporting robust research. We note that although our discussion summarizes measures taken by a sampling of Indigenous Peoples, each community ultimately is free to choose and require practices that meaningfully implement Indigenous research sovereignty in its own particular context. This expression of self-determination calls not only for a trusting and equitable relationship in which to understand and apply these practices, but also for Indigenous leadership in design and control of Indigenous research governance processes, both internally and in external, non-Indigenous environments that seek to or do include Indigenous Peoples.

### 4.1. Recognizing rights and interests

UNDRIP, the Nagoya Protocol, and the Global Indigenous Data Alliance have detailed numerous rights that Indigenous Peoples have in relation to data (United Nations General Assembly, 1993, 2007; UN Secretariat of the Convention on Biological Diversity, 2010; Hudson et al., 2023). Such broad rights to data address legal and ethical issues around storage, ownership, access, and consent (Kukutai and Taylor, 2016). In exercising research sovereignty to protect and advance their rights and interests, Indigenous Peoples in the US—most notably those with explicit status—have developed law and policy to set expectations for researchers and institutions working with their data.

#### 4.1.1. Sovereignty and self-determination

The documents analyzed require researchers to recognize the authority of Indigenous Peoples to control research involving their citizens, data, and resources both within and beyond their lands in alignment with their jurisdiction and ethics frameworks. Their jurisdiction is based on preexisting rights of sovereignty and self-determination (Sahota, 2007; Climate and Traditional Knowledges Workgroup (CTKW), 2014; Matson et al., 2021). To ensure that Indigenous aspirations are included throughout research, Indigenous Peoples are to be included in communications and decision-making from project inception to completion (Tribe 15). Indigenous Peoples have the authority to dictate how their data and knowledges are organized, stored, and managed (Tribes 4, 25, and 15). An implication of this codified right is the need for this authority to be reflected in the practices of researchers and within institutional policy. Research goals, agreements, and activities should be relevant and responsive to Indigenous needs, reflect Indigenous philosophies and practices, and remain collaborative to center Indigenous-defined objectives (Tribes 4 and 25).

#### 4.1.2. Assessment

Research with Indigenous Peoples requires assessment of collective risks and benefits. Risk assessment focuses on the protection of people, culture, and natural resources. Researchers show respect for participants, their families, and non-participating community members by clearly communicating relevant institutional policies that address collective risks, impacts, and protections. Researchers are to respect the social, economic, political, cultural, and spiritual epistemologies of Indigenous Peoples by upholding their rights to control and protect their knowledges and intellectual property (ISE, 2006; AIATSIS, 2012, 2020; Campbell et al., 2015; Tribe 8).

Research should maximize benefits while educating and empowering Indigenous Peoples (Tribe 4). This goal is exemplified in the Native Hawaiian “G.R.E.A.T. Research” (Governance, Re-consent, Education, Accountability, Transparency, Research priorities) framework for biobanking and biospecimen research (Tauali‘i et al., 2014). Implementation of “G.R.E.A.T. Research” requires researchers to ensure that their research goals are understood by the community, that findings are shared and not siloed, and that the community benefits from the collaboration. Benefits should be framed in collective terms from project inception to completion, and can include co-authorship, acknowledgment and attribution, intellectual property, and royalties or other monetary compensation (Tribe 25). Researchers also need to notify relevant Indigenous authorities of plans for commercialization and respect restrictions on commercialization (Tribes 18 and 4).

### 4.2. Governance of data

Many Indigenous authorities exert jurisdiction over people, places, issues, interests, and rights both within settler colonial defined geographic boundaries and beyond (Akwesasne Task Force on the Environment, 1996; South African San Institute, 2017; Around Him et al., 2019; Centre for Sami Health Research (CSHR), 2019; National Khoisan Council, 2019; Karuk-UC Berkeley Collaborative, 2020; Kuhn et al., 2020; Carroll et al., 2021, 2022; Hudson et al., 2021; Saunkeah et al., 2021; US Department of Agriculture Forest Service, 2023). This Indigenous conception of lands refers to physical lands and waters, including longitude and latitude data, as well as the environmental, ethical, and spiritual relationships embedded in these physical spaces (Liboiron, 2021). Researchers and institutions need to understand that Indigenous Peoples have the sovereign right to regulate all research (1) conducted on lands to which they relate, (2) that incorporates their knowledges, and (3) that involves their people (AIATSIS, 2012, 2020; Kukutai et al., 2023). In exercising research sovereignty, Indigenous Peoples in the US have developed governance mechanisms that define minimum expectations for researchers and institutions that hold and use Indigenous data.

#### 4.2.1. Jurisdiction/control

##### 4.2.1.1. Research process

All documents analyzed require proper authorization (e.g., permits, licenses, agreements) for research activities occurring within the jurisdiction of Indigenous Peoples and/or conducted with their members (Tribes 1, 4, and 19). It is a key exercise of research sovereignty for Indigenous Peoples to be final decision makers on project approvals (Tribe 10, 23, 2, and 5). Indigenous Peoples have the right to regulate research within their geographical boundaries and beyond when they have a cultural or intellectual property claim to materials or data (Tribes 22 and 1). Once a project is underway, researchers are required to share periodic updates on their research progress with the community (e.g., reviewing body, participating individuals, broader community; Tribes 1 and 19). Indigenous Peoples have the right to provide input on, and offer corrections to, data or findings being considered for publication (Tribes 25, 23, 8, 11, 5, and 2). Researchers have a corresponding responsibility to address concerns raised and make amendments as appropriate (Tribes 19 and 22). Per sovereignty principles, researchers are to comply with Indigenous Peoples' requests to withdraw from projects and/or return all samples (Tribes 23, 25, 8, and 11). Researchers need to understand that Indigenous authorities, being sovereign stewards of their data, may also deny, limit, or halt research (Tribes 12, 4, and 11). At the final stages of any research project, whether terminated or completed, samples, and data must be returned to the Indigenous community (Tribes 23, 25, and 24).

##### 4.2.1.2. Indigenous participants

Apart from regulating research on Indigenous lands and with Indigenous collectives, the legislation and policy surveyed protect Indigenous study participants as individuals (Tribes 19, 1, and 21). The documents call for researchers to comply with review processes that govern collection, storage, and publication of data to protect Indigenous persons from adverse effects and facilitate access to research benefits.

##### 4.2.1.3. Specimens, research materials, and data

The documents oblige researchers to respect Indigenous Peoples' values and regulations concerning confidentiality, privacy, anonymity, disaggregation, and protected information (Tribes 21 and 13). Institutional policies governing storage, access, use, and disposition of research samples, materials, and data must protect Indigenous Peoples and their individual members from improper uses, such as those by unsanctioned third-parties or for unauthorized commercial ends (Tribes 4, 24, and 21). Researchers should partner with Indigenous Peoples to design processes for data collection, storage, access, use, and disposition before, during, and after research projects, recognizing that Indigenous Peoples have the right to know and control how their data is being collected, stored, accessed, shared, and disposed of (Tribes 18, 4, 11, and 14).

##### 4.2.1.4. Publication/distribution

In the documents, Indigenous Peoples assert their right to review research data and findings being considered for publication in all formats, a claim that includes the authority to determine what can be released and to whom (Tribes 8, 11, 5, and 2). An implication of this right is the creation of complementary measures at research institutions to facilitate such review and implement such controls.

#### 4.2.2. Enforcement

Indigenous Peoples address potential harms by researchers and their institutions by adopting enforcement measures to support their legislation and policies. These enforcement tools include administrative actions effected through Indigenous government agencies as well as judicial penalties, fines, and orders issued by Indigenous courts. As such, researchers and their institutions need to be aware of applicable research laws and policies adopted by Indigenous Peoples.

##### 4.2.2.1. Administrative enforcement

Working with Indigenous Peoples is a privilege because Indigenous governments have primary authority over research conducted on their lands and with their members. As sovereigns, Indigenous governments can issue administrative sanctions against researchers who are accessing data or conducting research without authorization (Tribes 23, 14, and 24). Indigenous authorities can expel and bar researchers from their territories and require that all collected or generated data be returned (Tribes 8 and 11). In addition to bans and exclusions, administrative enforcement includes sending reports of violations to appropriate authorities (e.g., researchers' sponsors, funders, and licensing organizations, in addition to local, state and federal authorities; Tribes 8, 25, and 21).

##### 4.2.2.2. Judicial enforcement

Indigenous jurisdiction over research includes the right to address violations of law (e.g., unauthorized data collection) by researchers and institutions through Indigenous courts. Sanctions applied by Indigenous courts encompass civil and criminal judgments calling for fines, fees, and even jail time (Tribes 8, 25, 21, 23, 14, 24, 2, and 13). Judicial enforcement also includes equitable relief, a court-granted remedy that requires parties to act or refrain from performing particular acts. Such relief includes court orders to stop ongoing studies or to bar sponsors from funding further research with an Indigenous community (Tribes 23, 14, 24, 21, 2, and 13).

## 5. Indigenous research sovereignty: from principle to practice

The principles grounding our discussion of research sovereignty align among Indigenous Peoples globally, especially across similarly situated communities in Australia, Aotearoa New Zealand, and Canada. This article aims to move the conversation from principles to practices. We detail measures in Tables 1 and 2 to inform (1) Indigenous Peoples' efforts to design and revise governance mechanisms, laws, and policies, and (2) researchers and institutions as they implement policies, mechanisms, and practices to support Indigenous Peoples' research sovereignty.

**Table 1:** Recognizing Indigenous Peoples' rights and interests in research sovereignty: actions for researchers and institutions.

	**Actions for researchers**	**Actions for institutions**
**Sovereignty/self determination**		
Sovereignty/self determination	Recognize Indigenous Peoples' jurisdiction, control, and self-determination in research.	Engage local Indigenous Peoples to advise on creation and implementation of research policies and practices while incorporating Indigenous Peoples' rights and interests into institutional guidelines and policies through appropriate mechanisms (e.g., MOUs/MOAs/agreements; collective consent from Indigenous leadership for IRB approval of research involving Indigenous Peoples).
Ownership	Recognize Indigenous Peoples' ownership of their knowledge, data, information, and specimens.	Build recognition of Indigenous Peoples' ownership of research materials and data into administrative procedures and practices (e.g., required consultation, drafting of MOUs/MOAs/agreements/contracts).
Indigenous community worldviews	Center Indigenous Peoples' philosophies and community objectives while being culturally responsive.	Negotiate MOUs/MOAs/agreements/contracts that recognize and respect Indigenous Peoples' values, cultures, philosophies, and objectives.
Rights, interests, and priorities	Actively solicit Indigenous Peoples' research goals and priorities for incorporation throughout project lifecycle while respecting their restrictions.	Establish research MOUs/MOAs/agreements/contracts that reflect the values, interests, and agendas of participating Indigenous Peoples.
**Assessment of collective risks and benefits**		
Collective risks posed by research generally	Consider and plan for collective risks and impacts of research in collaboration with participating Indigenous Peoples throughout lifetime of the project.	Account for collective risks, impacts, and protections in policies governing research review, publication, data stewardship, while supporting innovation that aligns research with Indigenous Peoples' cultural, social, economic, and political wellbeing.
Risks to individual citizens/members, families	Understand, respect, and respond to perspectives of individual participants, participants' family members, and non-participating Indigenous community members in conceptualizing, conducting, and publishing research.	Develop research review, publication, and data stewardship policies and procedures that account for the perspectives of individual participants, participants' family members, and non-participating Indigenous community members in defining risks and protections.
Collective risks to culture/spirituality	Respect cultural values and traditions of Indigenous Peoples (as collectives), individual participants, participants' family members, and non-participating Indigenous community members in conceptualizing, conducting, and publishing research.	Develop research review, publication, and data stewardship policies that respect cultural values and traditions of Indigenous Peoples (as collectives), individual participants, participants' family members, and non-participating Indigenous community members.
Collective risks to Indigenous knowledges/intellectual property	Respect Indigenous Peoples' right to control and protect their knowledges and intellectual property.	Recognize Indigenous knowledges and cultural intellectual property rights in legal and policy infrastructure governing research and innovation.
Collective benefits of research generally	Ensure that benefits, as defined by participating Indigenous Peoples, are maximized throughout research.	Strengthen discipline appropriate research cultures and methodologies that are community engaged [e.g., Community Based Participatory Research (CBPR)] and develop research and funding infrastructures that are administratively responsive to Indigenous Peoples' understandings of research benefits.
Collective benefits from commercial use	Obtain consent from Indigenous Peoples for commercialization of research outputs and make arrangements for benefit sharing if communities agree to commercialization.	Co-develop with Indigenous Peoples a clear institution-wide policy position on commercialization of outputs from research involving Indigenous Peoples so they can negotiate benefits and prevent exploitation.

**Table 2:** Indigenous Peoples' governance of data in research sovereignty: actions for researchers and institutions.

**Themes**	**Actions for researchers**	**Actions for institutions**
**Jurisdiction/Control**		
**Research process**		
Issuance of permits	Comply with permitting requirements and community code provisions adopted by Indigenous Peoples.	Mandate that researchers comply with permitting requirements set by Indigenous Peoples.
Cancellations/revocations of permits	Acknowledge and respect Indigenous Peoples' rights of participation, withdrawal, and refusal with regard to research.	Develop research infrastructure that acknowledges Indigenous Peoples' rights of participation, withdrawal, and refusal with regard to research, including recognition of administrative actions by Indigenous research review mechanisms.
Jurisdiction	Recognize Indigenous Peoples' authority to control research conducted on their lands, but also anywhere beyond their geographical boundaries when projects involve their intellectual property, knowledges, cultures, and members/citizens.	Develop research infrastructure (including policies, guidance, and training) that acknowledges and upholds Indigenous Peoples' authority to control research within their lands, but also anywhere beyond their geographical boundaries when projects involve their intellectual property, knowledges, cultures, and members/citizens.
Research reports/updates	Provide progress reports to Indigenous Peoples through designated channels and seek community feedback at appropriate times throughout research.	Provide resources and training on culturally responsive engagement with Indigenous Peoples, including emphasis on administrative accountability to Indigenous research partners.
**Indigenous participants**		
Protection of participants	Comply with existing Indigenous review requirements for research, data collection, or publication involving individual Indigenous participants both on their lands and extending to protect members/citizens wherever they reside.	Ensure researchers are aware of (and research infrastructure accounts for) potentially distinct review requirements set by Indigenous Peoples for research, data collection, or publication involving individual Indigenous participants both on their lands and extending to wherever they reside.
**Data, specimens, and research materials**		
Privacy/confidentiality	Protect privacy of both Indigenous Peoples and individuals, as well as confidentiality of data and knowledge generated through research, by complying with Indigenous Peoples' expectations and regulations concerning sensitive and restricted data, specimens, and research materials.	Enact culturally responsive institutional policies recognizing the authority of Indigenous Peoples to protect individual and collective privacy as well as confidentiality of data and knowledge generated by research.
Storage/access/use/disposition	Collaborate with Indigenous Peoples to design processes for collection, storage, access, use, and disposition of research materials and data before, during, and after research (e.g., regular reporting on inventories and chain of custody).	Recognize in policies, procedures, trainings, and resources that Indigenous Peoples have the right to know and control how specimens, research materials, and data are collected, stored, accessed, and disposed of.
Third-party/future uses	Obtain formal permission from Indigenous Peoples regarding third party access and secondary/future uses of specimens, research materials, and data.	Establish procedures to notify Indigenous Peoples and researchers of requests for access to, and secondary use of, Indigenous Peoples' specimens, research materials, and data.
Return	Comply with Indigenous Peoples' requests to terminate studies and to return data and research materials.	Implement policies recognizing Indigenous Peoples' ownership of specimens, research materials, and data including rights to have data and research materials returned or disposed of upon request.
**Publication and dissemination**		
Review	Comply with Indigenous Peoples' requirements for review of research data or findings prior to publication.	Develop mechanisms and update publication standards to recognize and implement Indigenous Peoples' authority to review data from research being considered for publication or presentation in any format (e.g., reports, scholarly outputs, presentations, data summaries).
Input and modification	Address concerns and correct errors identified by Indigenous Peoples in reports, scholarly publications, and data summaries before they are published.	Develop mechanisms and update publication standards to recognize and incorporate Indigenous Peoples' processes for input into final versions of publications (e.g., reports, scholarly outputs, presentations, data summaries).
**Enforcement**		
**Administrative enforcement**		
Reporting violations	Understand that Indigenous authorities can report violations to researchers' sponsors, funders, and licensing organizations as well as other Indigenous communities and other local, state, and federal authorities.	Ensure administrative mechanisms exist to receive, handle, and respond to violations of Indigenous Peoples' research laws and policies.
Bans/exclusions	Understand that Indigenous authorities have the sovereign right to expel or ban from Indigenous lands researchers and institutions who violate Indigenous laws.	Provide education on key aspects of Indigenous sovereignty, including on the authority to expel or ban from Indigenous lands parties (e.g., researchers, institutions) who violate Indigenous laws.
**Judicial enforcement**		
Sanctions/equitable relief	Understand that researchers may face civil or criminal sanctions (e.g., fines/penalties) as well as equitable relief (e.g., injunctions) imposed by Indigenous Peoples' courts or any court with jurisdiction.	Provide education on key aspects of Indigenous sovereignty, including on authority to impose legal sanctions or restrictions through Indigenous courts or any court with jurisdiction.

The tables summarize recommendations for researchers and institutions to advance Indigenous Peoples' research sovereignty. Below is a discussion of strategies Indigenous Peoples and other institutions can implement.

### 5.1. Implement IDSov and IDGov principles at research institutions

Research institutions and sponsors need to move beyond recommending customized ethics guidelines for projects involving Indigenous Peoples, instead incorporating IDSov approaches (e.g., CARE Principles) into relevant features of research infrastructure such as project applications, contracting, IRB review, researcher training, metadata fields, data management and repository policies, funding requirements, and community engagement. This responsibility of aligning institutional policies and procedures with Indigenous self-determination is grounded in more than the motivation to protect “vulnerable” groups in research. It is founded on the deeper principle, running through UNDRIP, of restoring respectful, equitable relationships with Indigenous Peoples after centuries of unequal and exploitative engagements. Importantly, such implementation extends Indigenous Peoples' expectations around the responsible use of their data beyond the geographic, political, and social limits imposed by historical and ongoing colonial law and policy.

### 5.2. Adopt relevant and up to date governance

Indigenous Peoples need to ensure that existing governance documents are updated regularly to respond to changing technologies and data practices. For example, most documents analyzed employ approaches developed at the turn of the millennium mainly in response to advances in genetics. However, subsequent changes in research practice catalyzed by big data, open science, digital storage, and open data may call for further revisions to ensure that Indigenous Peoples' data remain under Indigenous control. Because updates to legislation often take time, research governance infrastructure should be robust enough to address current challenges yet flexible enough to respond to shifting trends in science.

### 5.3. Make governance materials available and accessible

Consistent compliance with Indigenous Peoples' research requirements calls for researchers and institutions to be aware of Indigenous Peoples' expectations. The need for such awareness is amplified by practice trends driven by big data and open science involving large scale pooling of data as well as collaboration among researchers and entities. Indigenous governments need to make research legislation and policy more widely accessible. For example, most documents analyzed for this article were obtained from third-party online sources compiled by non-Indigenous governments, universities, and other entities. To foster ready access to current requirements, Indigenous governments can host such information on their official websites or collaborate (regionally or nationally) to create vetted and regularly updated databases such as the International Compilation of Human Research Standards (US Department of Health and Human Services Office of Human Research Protections, 2022) and the Digital Publication of Tribal Laws Pilot Project (University of Wisconsin-Madison et al., 2020). Indigenous Peoples without formal research governance infrastructure can publish contacts and resources to inform researchers appropriately (US National Park Service, 2019; University of Arizona, n.d).

## 6. Conclusion

A key justification for Indigenous self-determination is the collective ability to respond to external currents driven by nation-state priorities and global trends. This pertains especially to the “data revolution”, which has outpaced effective regulation by governments, Indigenous and otherwise. Given this governance gap, Indigenous Peoples' sovereign actions in data governance are foundational. However, wide-ranging changes implicating a spectrum of data actors beyond Indigenous jurisdiction are needed to make data fair, accountable, and responsive to Indigenous Peoples' rights, interests, and responsibilities (White House Office of Science and Technology Policy, 2022).

## Data availability statement

The original contributions presented in the study are included in the article/Supplementary material, further inquiries can be directed to the corresponding author.

## Author contributions

IG: Conceptualization, Writing—original draft, Writing—review and editing. RS: Writing—original draft, Writing—review and editing. RP: Writing—original draft, Writing—review and editing. WC: Writing—original draft, Writing—review and editing. FMC-M: Writing—original draft, Writing—review and editing. JC: Writing—original draft, Writing—review and editing. CC: Writing—original draft, Writing—review and editing. DD-C: Writing—original draft, Writing—review and editing. AF: Writing—original draft, Writing—review and editing. DH: Writing—original draft, Writing—review and editing. VH: Writing—original draft, Writing—review and editing. MH: Writing—original draft, Writing—review and editing. MBJ: Writing—original draft, Writing—review and editing. LLJ: Writing—original draft, Writing—review and editing. AM: Writing—original draft, Writing—review and editing. JY: Writing—original draft, Writing—review and editing. NAG: Writing—original draft, Writing—review and editing, Conceptualization. SRC: Conceptualization, Writing—original draft, Writing—review and editing.
