# Supplementary material for: Indigenous Peoples and research: self-determination in research governance
Source: Front Res Metr Anal. 2023 Nov 15;8:1272318. doi: 10.3389/frma.2023.1272318 (PMC10685893; doi:10.3389/frma.2023.1272318)
Supplement: Supplementary file 1 [file Data_Sheet_1.PDF]

Supplementary Table 1. Examples of Text from Indigenous Research Governance Documents

Themes	Summary	Text from Indigenous Research Governance Documents
**CARE SUBPRINCIPLE ("AUTHORITY TO CONTROL"): *RECOGNIZING RIGHTS AND INTERESTS***		
**Sovereignty / Self-determination:** *The documents include assertions of Indigenous sovereignty/self-determination as the foundation for research regulation. These statements often include the Indigenous government's "inherent" right to regulate affairs—research included—within their territories and with their citizens.*		
1) Sovereignty / self-determination	Indigenous Peoples have authority to control research on their lands and within their jurisdiction, based on their preexisting (i.e., precolonial) rights to sovereignty and self-determination.	WHEREAS the [Tribe] by and through the Tribal Council, has the inherent sovereign authority to regulate the conduct and activities on all lands within the jurisdiction of the Tribe ... (Tribe 4)<br><br>Sovereignty and tribal law are the key concepts researchers should keep in mind when approaching the [Tribe] with a research request. (Tribe 1)
2) Ownership	Indigenous Peoples have sovereign rights of ownership over knowledge and information derived from their territories, communities, and members; uses of such data are subject to Tribal control and require collaboration.	The Nation has a right to ownership of the work product created by the research or researcher. (Tribe 8)<br><br>The … Tribe reserves the right to: ... 4. Assert full ownership or grant co-authorship of products or research findings. (Tribe 10)
3) Indigenous community worldviews	Research conducted with Indigenous Peoples should be responsive and accountable to the culture, values, and world views of the particular community involved.	A good research agreement will be developed to ensure that studies proceed in a manner that is both culturally sensitive and relevant to the participants and community. (Tribe 25)<br><br>The purposes of the ERB are to assure that research and publication activities: (1) Are consistent with the cultural, health and education goals and objectives of [the Tribe]. (Tribe 12)
4) Rights, interests, and priorities	All stages of the research process must involve Indigenous Peoples to support the rights, advance the interests, and reflect the priorities of their communities.	The process of developing community-based and culturally relevant research should directly include the tribe from the studies [*sic*] inception and supports [*sic*] a tribal agenda (plus whenever possible include local Native American investigators). (Tribe 3)<br><br>An explicit aim of the second … research legislation was to ensure that research conducted within the … Nation is beneficial to the tribe, community-based and consistent with [the Tribe's] values, priorities and concerns. (Tribe 1)
**Assessment of Collective Risks and Benefits:** *Documents describe two areas of research regulation in which Indigenous governments occupy a unique position to exercise their sovereignty: the determination of (a) collective risks and (b) collective benefits.*		

Themes	Summary	Text from Indigenous Research Governance Documents
1) Collective risks posed by research generally	Research regulation protects the people, culture, and natural resources of the Indigenous community from risks (e.g., physical, psychological, cultural, social, economic, political) associated with research.	[T]he following information shall be provided by an applicant in support of an application for a permit: ... (4) Risks associated with or inherent in the research, including risks to the physical or psychological well-being of individual human subjects or participants and risks of deleterious impact on the cultural, social, economic, or political well-being of the community. (Tribe 8)<br><br>The … Tribal Council has taken steps to protect and preserve [the Tribe's] people from harm in research by authorizing the [IRB] to provide oversight and monitoring of all research conducted within the [Nation's] borders. (Tribe 1)
2) Collective risks to individual citizens-members / families	Individual participants, Indigenous community members, and their families should be protected from unauthorized, insensitive, inaccurate, stereotypical, or harmful uses of data.	The purpose of this Act is to (a) protect the people, culture, and natural resources of the Tribe and the Tribe's future generations from unauthorized scientific research. (Tribe 4)<br><br>The [Department of Health] shall assure the safety, well-being, confidentiality, dignity, and integrity of the [Tribe's] people and all American Indians served by the [Department of Health] when participating in research. (Tribe 22)
3) Collective risks to culture / spirituality	Research should be culturally responsive, respecting and protecting Indigenous Peoples' sacred traditions and culture for future generations.	Principle of Respect: This principle recognizes the necessity for researchers to respect the integrity, morality, and spirituality of the culture. (Tribe 4)<br><br>The Tribal Council also has a fundamental responsibility to protect and preserve the culture of the Nation and to ensure that the IRB permitted activities are conducted in a way that does no harm to the culture of the Nation. (Tribe 18)
4) Collective risks to Indigenous knowledges / intellectual property	Indigenous Peoples have the right to protect and control their traditional knowledges and other forms of intellectual property.	The Tribe finds that it is in its best interests to establish a research review mechanism to prevent the continued [research] abuses, to protect the people's traditional knowledge and properties, and thereby to ensure our rights to continue to practice traditional lifeways for our long term survival. (Tribe 4)<br><br>All references to or descriptions of [tribal] culture, history, or language the ERB deems sacred, inaccurate, or in violation of [the Tribe’s] intellectual property rights must be removed or corrected prior to publication. (Tribe 12)

Themes	Summary	Text from Indigenous Research Governance Documents
5) Collective benefits of research generally	Indigenous Peoples' regulation of research ensures the maximization of direct benefits to the community and its members.	Just compensation or fair return includes but is not limited to: obtaining copies of the research findings, authorship, co-authorship or acknowledgment, royalties, fair monetary compensation, copyright, patent, trademark, compensation for expenses incurred in reviewing/advising researchers, coverage of training/education or outreach expenses or other forms of compensation. (Tribe 25)<br><br>The research should be of direct benefit to the Tribe and its Members, and the risks associated with the research should be less significant than the benefits to be gained. (Tribe 2)
6) Collective benefits from commercial use	Any plans for commercialization of research findings, materials, specimens or other data must be approved by the Indigenous community prior to the start of research, including any benefit sharing arrangements.	No entity may seek to patent or commercialize any biological materials obtained from the Tribe, from the Tribe's jurisdiction, or under the authority of the Tribe including but not limited to, genetic samples, any copies of the original genetic samples, any cell lines derived from collected materials, and data derived from these samples. (Tribe 4)<br><br>What are the plans (pre, duration, post-project) for publication or commercialization of the product or research findings? (Tribe 18)
**CARE SUBPRINCIPLE (“AUTHORITY TO CONTROL”):<br>*GOVERNANCE OF DATA***		
**Jurisdiction / Control:** *Indigenous Peoples exercise their sovereignty over research by controlling activities at the points listed below.*		
1) Research process		
Issuance of permits	Any entity undertaking research on Indigenous lands or with Indigenous persons must first obtain a permit from appropriate Indigenous authorities.	All persons proposing to conduct on-site research within [the Tribe’s territory] shall first apply for and obtain a permit from the [Office of Cultural Affairs]. (Tribe 9)<br><br>There is hereby created the … Review Board, whose purpose is to review all proposals (notwithstanding other IRB approvals) for human research which will occur within the territorial jurisdiction of the … Nation or which otherwise concerns [the community’s] individuals as an identifiable group, issue permits for those projects which are consistent with the terms and intent of this Code, and, as appropriate, review and approve the results of such studies before publication. (Tribe 1)

Themes	Summary	Text from Indigenous Research Governance Documents
Cancellations / revocations of permits	Indigenous Peoples are not obligated to participate in any research activity and can withdraw their consent at any time if the terms of approval are being violated, including if the research is deemed harmful to individuals or the community.	The permit issued pursuant to this Chapter is conditional and may be canceled at any time if it appears that the individual, corporation, agency or institution conducting the study, survey or research project is deviating or has deviated from the study design approved in the granting of the permit, or from provisions of the required underlying agreement upon which issuance of the permit is based. (Tribe 23)<br><br>The … Nation … reserves the right to: ... 3. Withdraw approval for projects. When this is done, the … Nation … will explain the rationale for withdrawing approval and explain why this project or the release of data is deemed to be harmful to individuals or the community as a whole. (Tribe 25)
Jurisdiction	Indigenous Peoples have the right to regulate all research conducted on their lands, and involving their intellectual property, culture, members/citizens, or other resources wherever located.	The [Tribal] IRB shall have the authority to approve or deny all such research, data collection, or participant recruitment proposed to occur within the boundaries of the [Tribe's territory]. (Tribe 22)<br><br>[This Code] may also be enforceable outside the jurisdiction of the [Tribe] as applicable law permits with respect to research conducted on the [Tribe's] lands or research using materials as to which the [Tribe] has claim of ownership. (Tribe 14)
Research reports / updates	Indigenous Peoples have the authority to require progress reports throughout the lifetime of the project, including on results and findings.	Describe how frequently and in what manner aggregate data and progress reports will be shared with the [Nation]. Describe communication strategies that will be used to present aggregate data to the community at large. (Tribe 25)<br><br>At least one (1) copy of all interim and/or progress reports, and of the final report resulting from the study, surveyor research project, shall be furnished to the office of the Tribal Chairman. (Tribe 23)
2) Indigenous participants		
Protection of participants	Indigenous Peoples exert jurisdiction over research both within their lands and involving their members / citizens wherever they reside.	The Tribal Council ... declare the purpose of this Ordinance as follows: ... (h) To establish and provide a statutory basis for a process to review and govern any research, collection, database, or publication undertaken with any tribal citizen specifically because they are members of [the Tribe]. (Tribe 19)<br><br>There is hereby created the … Review

Themes	Summary	Text from Indigenous Research Governance Documents
		Board, whose purpose is to review all proposals (notwithstanding other IRB approvals) for human research which will occur within the territorial jurisdiction of the [ ] Nation or which otherwise concerns [the community's] individuals as an identifiable group. (Tribe 1)
3) Specimens, research materials, and data		
Privacy / confidentiality	The privacy of both Indigenous Peoples and individuals should be protected, and the confidentiality of data, specimens, and knowledges generated through research should be preserved.	SPECIMENS: The applicant shall also identify those persons or entities that will have access to the biological specimens during Research and shall state whether such specimens will be collected with, or de-linked from, personal identifiers of the donor(s). (Tribe 21)<br><br>DATA: Principle of Confidentiality: This principle recognizes that the Tribe and local communities, at their sole discretion, have the right to exclude from publication and/or to have kept confidential any information concerning their culture, traditions, mythologies, or spiritual beliefs. Furthermore, researchers and other potential users shall guarantee such privacy and confidentiality. (Tribe 24)
Storage / access / use / disposition	Indigenous Peoples can require up-to-date records specifying location, data type, provenance details, accessibility restrictions, and confidentiality requirements of all stored data and specimens.	SPECIMENS: If the Tribe permits any biological samples to be stored in any other locations, the Tribe will maintain jurisdiction over the samples, and the researcher shall maintain at all times a complete list thereof. The list shall include a description of the sample or data [...] (Tribe 4)<br><br>DATA: The … Tribe reserves the right to: … Require deposit of raw materials or data, working papers or products in a tribally designated repository, with specific safeguards to preserve confidentiality. (Tribe 18)
Third-party / future uses	Samples, research materials, and data are not to be shared with third parties or used for other projects without approval of Indigenous authorities or consent of Indigenous participants.	SPECIMENS: If the Research involves biological specimens, the applicant shall state whether biological specimens will be stored by the researcher after Research is completed, for possible use in future research or for other reasons, or whether biological specimens will be returned to the donor(s) or destroyed. (Tribe 21)<br><br>DATA: The data will not at any future time be

Themes	Summary	Text from Indigenous Research Governance Documents
		repurposed by the principal investigator or academic sponsor to any other use outside the stated purpose of the original study application, without first obtaining specific permission from the … Research Review Board. (Tribe 11)
Return	When research is completed or if approvals are withdrawn, samples and data must be returned to Indigenous Peoples.	SPECIMENS: The Tribe may, at any time, decide to withdraw from the research project or any portion thereof, and request the return of all biological samples. (Tribe 4)<br><br>DATA: In the case of withdrawal of approval by the Tribe, all information and copies of data must be returned to the Tribe. (Tribe 4)
4) Publication and dissemination		
Review	Research data and findings must be reviewed and approved by Indigenous Peoples before they are published or presented in any format or forum.	Any permission to publish must be granted by the IRB prior to publication. (Tribe 8)<br><br>Any proposed publication-content or presentation-content will be offered for tribal review before publication or presentation. (Tribe 11)
Input and modification	Indigenous Peoples have the right to review, provide input, recommend modifications, and place limitations or prohibitions on research outputs, while researchers are to respond to such recommendations and restrictions with appropriate recognition of Indigenous sovereignty.	All non-Tribal parties wishing to carry out research, projects, and activities pertaining to [the Tribe's] traditional culture must submit a proposal ... that will include the following: (8) A description of how the Tribe will have an opportunity to review the project or activity results before the final products are published or otherwise used, and how the Tribe may offer input into this material. (Tribe 5)<br><br>Principle of Confidentiality: This principle recognizes that the Tribe and any human research subjects, at its/their sole discretion, have the right to exclude from publication and/or to have kept confidential, any information including information concerning themselves, their health, or their culture, traditional knowledge, traditions, mythologies, or spiritual beliefs, and all researchers and other potential users shall guarantee such confidentiality. (Tribe 2)
**Enforcement:** *The documents describe mechanisms that Indigenous authorities use to enforce requirements for conducting research within their jurisdictions.*		
1) Administrative enforcement		
Bans / exclusions	Persons or institutions violating Indigenous research codes and policies may be expelled and/or banned from Indigenous lands,	Any outside agency or researcher conducting research within the jurisdiction of the Nation without a permit or otherwise in violation of this Chapter shall be subject to exclusion

Themes	Summary	Text from Indigenous Research Governance Documents
	restricted from contacting community participants, and denied future requests for partnership.	from the Nation in accordance with the Nation's laws governing removal and exclusion. (Tribe 8)<br><br>The … Nation … reserves the right to: ... (4) Deny researchers the opportunity to conduct research in the communities of the [ ] Nation … . In addition, other researchers or scientists from the same research institution may be denied any future access to the community. (Tribe 25)
Reporting violations	Violations of Indigenous research codes and policies may be reported to researchers' sponsors, funders, and licensing organizations as well as other Indigenous, local, state, and federal authorities.	If a civil suit is filed to enforce this Code, notice shall be given to the research project's sponsoring organization and/or funding source as well as to the professional organization or licensing agency of the person conducting the Research. (Tribe 21)<br><br>The researcher's funding source may be enjoined from any further research activities and the Nation may report the researcher and/or their funding source's actions to other tribes. (Tribe 13)
2) Judicial enforcement		
Sanctions and equitable relief	Indigenous Peoples have the right, as sovereigns, to impose legal sanctions through their courts or bring an action before any court with jurisdiction against researchers or institutions for violating research laws and policies.	Any person, over whom the Tribe may assert criminal jurisdiction, who knowingly violates or counsels, solicits, or employs any other person to violate any section of this Code, or any condition of limitation of a permit issued under this Code, shall be guilty of a criminal offense. Each criminal offense shall be punishable by restitution, community service, or a fine not to exceed $10,000, imprisonment in the tribal jail for not more than one year, or any combination of these penalties. Criminal offenders may also be subject to civil penalties and damages set forth in this Code. (Tribe 2)<br><br>Any person attempting to conduct research not specifically requested or contracted for by the Tribes or permitted pursuant to provisions of this Chapter shall be subject to any and all civil or remedies available pursuant to the Tribal Code of the Tribes and the laws of the State… . (Tribe 13)
