# Supplementary material for: Indigenous Peoples and research: self-determination in research governance
Source: Front Res Metr Anal. 2023 Nov 15;8:1272318. doi: 10.3389/frma.2023.1272318 (PMC10685893; doi:10.3389/frma.2023.1272318)
Supplement: Supplementary file 2 [file Data_Sheet_2.PDF]

Supplementary Table 2: US Indigenous Research Governance Documents Reviewed

Indigenous People	Document/Resource	Online Source
**Aleut Community of St. Paul Island**	Tribal Code: Intellectual Property, Photographing and Videotaping, Reproduction and Publishing Research Requirements (adopted: 2008; amended: 2010)	https://aleut.tribal.codes/ATC/7.30
**Cherokee Nation**	Tribal Research (updated: 2019)	https://irb.cherokee.org/media/zaulx5iz/tribal-reserach.pdf
	Renewal Guide (updated: 2019)	https://irb.cherokee.org/media/hj2nm2hi/renewal-guide.pdf
	Renewal Application (updated: 2019)	https://irb.cherokee.org/media/0bud1wq4/renewal-application.pdf
	Proposal Application (updated: 2019)	https://irb.cherokee.org/media/z1ali4y5/proposal-application.pdf
	Lay Summary Guide (updated: 2019)	https://irb.cherokee.org/media/abgdmvuz/lay-summary-guide.pdf
	Institutional Review Board (IRB) Overview (updated: 2023)	https://irb.cherokee.org/
	Informed Consent Guidance (updated: 2019)	https://irb.cherokee.org/media/vwdpetyi/informed-consent-guidance.pdf
	Helicopter Research (updated: 2019)	https://irb.cherokee.org/media/rkknqeww/helicopter-research.pdf
	Guidance on Study Closure (updated: 2019)	https://irb.cherokee.org/media/jezhjftz/guidance-on-study-closure.pdf
	Defining a Tribal Sponsor (updated: 2019)	https://irb.cherokee.org/media/qdih1lnf/tribal-sponsor.pdf
**Eastern Band of Cherokee**	Protocol with Minors Review Form (effective: 2003; revised: 2013)	https://phhs.ebci-nsn.gov/medical-institutional-review-board/

Supplementary Table 2: US Indigenous Research Governance Documents Reviewed

Indigenous People	Document/Resource	Online Source
	Protocol Application Form (2021)	https://phhs.ebci-nsn.gov/wp-content/uploads/2022/01/MIRB-Protocol-Submission-Form-002.pdf
	Medical Institutional Review Board (MIRB) Overview (n.d.)	https://phhs.ebci-nsn.gov/medical-institutional-review-board/#:~:text=The%20Eastern%20Band%20of%20Cherokee%20Indians%20has%20complete%20and%20final,that%20any%20research%20performed%20has
	Institutional Review Board (IRB) Flow Chart (2022)	https://phhs.ebci-nsn.gov/wp-content/uploads/2022/01/IRB-Flow-Chart.pdf
	Instructions: Protocol Submission (2018)	https://phhs.ebci-nsn.gov/wp-content/uploads/2021/06/MIRBInstructions-Protocol-Submission-2018.pdf
	Informed Consent Checklist (2018)	https://phhs.ebci-nsn.gov/wp-content/uploads/2021/06/MIRB-IC-Checklist-2018.pdf
**Chickasaw Nation**	Division of Health Policy: Release & Dissemination of Research Results & Findings (Effective: 1994; Review: 2020	https://chicresources.net/getattachment/Community-Health-Wellness/Chickasaw-Health-Research/Institutional-Review-Board/ADM-1411-Dissemination-of-Findings_2017_Final.pdf.aspx?lang=en-US
	Division of Health Policy: Institutional Review Board (effective: 1994; review: 2016)	https://chicresources.net/getattachment/Community-Health-Wellness/Chickasaw-Health-Research/Institutional-Review-Board/ADM-1410-CNDH-IRB_2017_Final.pdf.aspx?lang=en-US

Supplementary Table 2: US Indigenous Research Governance Documents Reviewed

Indigenous People	Document/Resource	Online Source
	Division of Health Policy: Participant Rights Pertaining to Research (effective: 1994; review: 2020)	https://chicresources.net/getattachment/Community-Health-Wellness/Chickasaw-Health-Research/Institutional-Review-Board/ADM-1401-Participants-Rights_2017_Final.pdf.aspx?lang=en-US
	Division of Health Policy: Participation in Research (effective: 1994; review: 2020)	https://chicresources.net/getattachment/Community-Health-Wellness/Chickasaw-Health-Research/Institutional-Review-Board/ADM-1400-Participation-in-Research_2017_Final.pdf.aspx?lang=en-US
**Colorado River Indian Tribes**	Human and Cultural Research Code (amended: 2020)	https://naair.arizona.edu/sites/default/files/CRIT_Human%20and%20Cultural%20Research_Final%20Published_02062020.pdf
**Confederated Tribes of the Colville Reservation**	Research Regulation (1981)	https://thorpe.law.ou.edu/archives/colville/CHPT6-6.html
**Confederated Tribes of the Coos, Lower Umpqua and Siuslaw**	Research Regulation (2002)	https://ctclusi.org/wp-content/uploads/2020/12/Ch-1-10-Research-Regulation.pdf
**Confederated Tribes of Siletz Indians**	Research Ordinance (enacted: 2000; amended: 2001; amended: 2005)	https://www.ctsi.nsn.us/wp-content/uploads/2020/12/Research-Ordinance-09-16-2005.pdf
**Gila River Indian Community**	Medical and Health Care Research Ordinance (2009)	https://naair.arizona.edu/sites/default/files/gila_river_indian_ord_gr-05-09_title_17_chapter_9_0.pdf

Supplementary Table 2: US Indigenous Research Governance Documents Reviewed

Indigenous People	Document/Resource	Online Source
**Ho-Chunk Nation**	Tribal Research Code (enacted: 2005; amended: 2005)	https://ho-chunknation.com/wp-content/uploads/2019/10/3HCC3-Tribal-Research-Code-05.05.05.pdf
**Hopi Nation**	Protocol for Research, Publication and Recordings: Motion, Visual, Sound, Multimedia and other Mechanical Devices (n.d.)	https://www.hopi-nsn.gov/wp-content/uploads/2021/09/HCPO-Research-Protocol.REVISED.2021.pdf
	Intellectual Property Rights (n.d.)	https://www8.nau.edu/hcpo-p/intellectPropRights.html
**Karuk Tribe**	Protocol on Karuk Tribe's Intellectual Property Rights Research, Publication and Recordings (adopted: 2014; amended: 2015)	https://sipnuuk.karuk.us/system/files/atoms/file/ATALM17_KTResearchProtocol.pdf
**Mandan, Hidatsa, and Arikara Nation (Three Affiliated Tribes)**	Research Code (n.d)	NA
**Muscogee (Creek) Nation**	Institutional Review Board (IRB) Code (2010)	https://www.creeksupremecourt.com/wp-content/uploads/T22-NCA10-124.pdf
**Navajo Nation**	Human Research Code (adopted: 1995; amended: 2002)	https://www.nnols.org/navajo-nation-code/
**Nez Perce Tribe**	Research Regulation Ordinance (n.d.)	https://www.nezperce.org//wp-content/uploads/2019/01/research-permit-form.pdf
**Oglala Sioux**	Research Review Board (RRB) Brochure (n.d.)	https://www.crcaih.org/assets/TribalPartner/OSTRRB_BrochureLB.pdf
	Presentation: Research Review Board: Exercising our Sovereignty (n.d)	NA

Supplementary Table 2: US Indigenous Research Governance Documents Reviewed

Indigenous People	Document/Resource	Online Source
	Research Review Board (RRB) Flyer (n.d)	https://www.crcaih.org/assets/TribalPartner/OST_importance_research_Final.pdf
	Approval Letter (2015)	NA
**Pascua Yaqui Tribe**	Regulatory Code (2008)	https://www.pascuayaqui-nsn.gov/wp-content/legacy-tribalcodes/index.html
**San Carlos Apache**	Procedures for Research Activity and Recording (n.d)	https://tribalinformationexchange.org/wp-content/uploads/2019/03/San-Carlos-Apache-Elder's-Cultural-Advisory-Council-Procedures-for-Research-Activity-and-Recording.pdf
**Seneca Nation of Indians**	Protocol for Review of Scientific Research Proposals on Territories of the Seneca Nation of Indians (n.d.)	NA
**Sisseton Wahpeton Oyate Tribe**	Research Code (n.d)	https://www.swo-nsn.gov/wp-content/uploads/Chapter-77-ResearchCode-FINAL-V15.pdf
**Tohono O'odham Nation**	Research Code (2013)	https://www.tolc-nsn.gov/docs/Title17Ch8.pdf
**Turtle Mountain Band of Chippewa Indians**	Research Protection Act (2014)	https://tnrg.org/research-review-board
**United Houma Nation**	Institutional Review Board (Research Committee) Ordinance (n.d.)	NA
**White Earth Nation**	Research Code (2017)	NA
	Revised/Codified Research Review Board Policy/Procedure (2018)	NA
